# Canalization effect in the coagulation cascade and the interindividual variability of oral anticoagulant response. a simulation Study

**DOI:** 10.1186/1742-4682-8-37

**Published:** 2011-10-12

**Authors:** Alexandru D Corlan, John Ross

**Affiliations:** 1 University Emergency Hospital, 169 Spl Independentei, Bucharest, Romania; 2 'Carol Davila' University of Medicine and Pharmacy, 21 Dionisie Lupu, Bucharest, Romania; 3 Chemistry Department, Stanford University, CA, USA

**Keywords:** Interindividual variability, stochastic model, oral anticoagulant treatment, INR, coagulation cascade, canalization

## Abstract

**Background:**

Increasing the predictability and reducing the rate of side effects of oral anticoagulant treatment (OAT) requires further clarification of the cause of about 50% of the interindividual variability of OAT response that is currently unaccounted for. We explore numerically the hypothesis that the effect of the interindividual expression variability of coagulation proteins, which does not usually result in a variability of the coagulation times in untreated subjects, is unmasked by OAT.

**Results:**

We developed a stochastic variant of the Hockin-Mann model of the tissue factor coagulation pathway, using literature data for the variability of coagulation protein levels in the blood of normal subjects. We simulated *in vitro *coagulation and estimated the Prothrombin Time and the INR across a model population. In a model of untreated subjects a "canalization effect" can be observed in that a coefficient of variation of up to 33% of each protein level results in a simulated INR of 1 with a clinically irrelevant dispersion of 0.12. When the mean and the standard deviation of vitamin-K dependent protein levels were reduced by 80%, corresponding to the usual Warfarin treatment intensity, the simulated INR was 2.98 ± 0.48, a clinically relevant dispersion, corresponding to a reduction of the canalization effect.

Then we combined the Hockin-Mann stochastic model with our previously published model of population response to Warfarin, that takes into account the genetical and the phenotypical variability of Warfarin pharmacokinetics and pharmacodynamics. We used the combined model to evaluate the coagulation protein variability effect on the variability of the Warfarin dose required to reach an INR target of 2.5. The dose variance when removing the coagulation protein variability was 30% lower. The dose was mostly related to the pretreatment levels of factors VII, X, and the tissue factor pathway inhibitor (TFPI).

**Conclusions:**

It may be worth exploring in experimental studies whether the pretreatment levels of coagulation proteins, in particular VII, X and TFPI, are predictors of the individual warfarin dose, even though, maybe due to a canalization-type effect, their effect on the INR variance in untreated subjects appears low.

## Background

There is a continuing need to identify measurable causes of the substantial variability of the individual response to oral anticoagulants, such as warfarin or acenocoumarol. Finding such causes is required to improve predictability and consequently reduce the high levels of morbidity and mortality associated to these treatments. Together with anthropometric parameters, the known polymorphisms of CYP2C9 and VKORC1 enzymes currently account only for about 50% of the dose variability [1-3].

The relation between measurable sources of variability and the variability of the patient response to treatment is explained by the complex but extensively explored warfarin pharmacokinetics/pharmacodynamics (PK/PD).

In short, Warfarin is absorbed from the gut completely, then it blocks irreversibly the Vitamin-K epoxyde reductase complex 1 (VKORC1), an enzyme found mostly in the liver. It is eliminated by other liver enzymes, from the cytochrome P-450 family, mostly by CYP450-2C9. The blocking of the VKORC1 results in a depletion of reduced Vitamin-K that is oxidised by the *γ*-glutamyl-carboxylase enzyme (GGCX) symultaneously with the *γ*-carboxylation of the glutamate residues of protein precursors of coagulation factors II,VII,IX and X. The *γ*-carboxylation is necessary for their biological activity.

The variability of this PK/PD process can be due to: genetic mutations of CYP2C9, VKORC1 and GGCX; mutations in the introns, exons or flanking sequences of these enzymes resulting in different phenotypical expression; variability in intake, transport and metabolism of vitamin-K; the size of the various organism compartments and in particular of the liver; the general synthesis capacity of the liver; simultaneous administration of drugs and foods that interact with various stages of the process, for example by inducing or repressing the expression of CYP450 enzymes.

The synthesis rate of coagulation factors, and their biological activity is also variable due to a number of mutations affecting both the structure and the expression rate of the precursors. The elimination rate is probably influenced by the general activation rate of the coagulation cascade in the bloodstream that also presents interindividual variability.

One potential cause of the response variability that has not been generally considered is that due to the genetic polymorphism and phenotypical expression of the proteins that intervene in the coagulation cascade. Their effect on the coagulation time, for example on the prothrombin time and its standardised equivalent--the International Normalised Ratio (INR), is low in the healthy population. Here we argue, based on a stochastic version of a well studied model of the extrinsic pathway of the coagulation cascade [4], that this effect should be low only in untreated subjects.

The *canalization effect*, [5,6] is the relative robustness of key physiologic parameters, features and processes despite wide genetic and phenotypical variability of other factors, such as expression levels of proteins.

We believe this could be the case with the coagulation cascade: while there is substantial interindividual variability in the biological level of coagulation proteins, the cascade is organised such that the key blood coagulation time parameter is maintained within very narrow limits across the healthy population.

This would also explain why a very substantial reduction of the level of vitamin-K dependent factors (II,VII,IX,X) needs to be obtained in order to observe a clinically relevant prolongation of the prothrombin time.

However, our simulations show that once this reduction is achieved, the canalization phenomenon is also supressed and the interindividual variability of the expression of coagulation proteins in treated subjects becomes manifest and needs to be corrected by adjusting the Warfarin dose.

## Methods

The Hocking Mann model [4] is a system of ordinary differential equations that describe the kinetics of the sythesis of active thrombin once tissue factor is added to a sample of blood. It corresponds to the process that takes place in a test tube when the prothrombin time is measured.

We developed a stochastic version of the Hockin-Mann model of the tissue factor (extrinsic) pathway kinetics by replacing, in the equations from [4] the coefficients corresponding to the initial levels of factors II, V, VII, VIIa, VIII, IX, X, XI, antithrombin-III and tissue factor pathway inhibitor (TFPI) with normally distributed, independent, random variables. The coefficient of variation for each variable was estimated based on direct experimental determinations available in the literature (see Table 1).

**Table 1:** Literature data on the dispersion of the coagulation protein levels

protein	mean	sd	unit	**c.v**. (%)	reference
II (prothrombin)	99.6	11.9	%	11.95	Yamagishi 2010 [20]

V (proaccelerin)	105.4	33.6	%	31.88	Yamagishi 2010 [20]

VII (proconvertin)	112.0	30.0	%	26.78	Folsom 1997 [21]
	130.0	33.0	%	25.38	Cushman 1996 [13]
	98.0	20.7	%	21.12	Feng 2000 [12]^1^, non-cvd
	99.5	26.2	%	26.33	Feng 2000 [12]^1^, non-thrombotic cvd
	93.6	31.7	%	33.87	Feng 2000 [12]^1^, thrombotic cvd

VIII	120.0	37.0	%	30.83	Cushman 1996 [13]
	127.0	40.0	%	31.50	Folsom 1997 [21]

IX	88.5	29.3	%	33.11	Yamagishi 2010 [20]

X (prothrombinase)	102.9	25.8	%	25.07	Yamagishi 2010 [20]

XI	84.1	19.7	%	23.42	Yamagishi 2010 [20]

Antithrombin-III	109.0	20.0	%	18.35	Folsom 1997 [21]

TFPI	36.4	12.8	ng/ml	35.16	Zakai 2010 [22]

Literature experimental data on the dispersion of coagulation protein biological levels in the general population, as used in our simulations. In each case we computed C.V., the coefficient of variation, that is the ratio of the reported sd (standard deviation) to the reported mean. The unit used in most reports is a percentage from a standard laboratory value that is taken as 100% and is assumed close to the population average.

Values marked with '^1^' were estimated from the standard error of the mean and the number of cases from each groups of cvd (cardiovascular disease) patients.

We used a 5nM concentration for the initial level of the tissue factor, consistent with the conditions used when measuring the prothrombin time. We computed a simulated prothrombin time as the reaction time needed for activated factor II to reach a concentration of 20 nM.

From the results we computed the equivalent of the prothrombin time for each realisation and the mean normal prothrombin time (MNPT), as in [7], by averaging the normal prothrombin times. The INR (International Normalised Ratio) of a blood sample is the ratio of the prothrombin time for that sample to the MNPT.

As previously described in reference [8] this approach, using the Hockin-Mann model, leads to simulated results that are consistent with the experimentally observed relation between the INR and the vitamin K dependent coagulation factor levels as reported in reference [9].

We simulated this model with a Monte Carlo implementation, by sampling the space of the variables and computing the kinetics of each of the species as proposed by Gillespie [10]. Each realisation corresponds to a simulated individual, and the whole dataset represents a simulation of the sample of the normal population.

The presence of stable anticoagulant treatment was simulated by uniformly reducing the initial levels of factors II, VII (and VIIa), IX and X down to 20%. The proportional reduction was applied to each case in the randomly generated set, thus resulting in the reduction of both the mean and the standard deviation of the initial values of each variable.

We then combined the stochastic Hockin-Mann model with our 2008 stochastic model of warfarin pharmacodynamics [11], which is a detailed simulation of the kinetics of warfarin absorption, elimination, binding to VKORC1; vitamin K cycle; coagulation factor synthesis, *γ*-carboxylation, release and elimination. It is a set of ordinary differential equations with stochastic coefficients that represent interindividual variabilities.

This model was tuned with data in a population of mostly Caucasian adults and is able to reproduce the dynamics of INR response and dose variability as found in the literature.

In the combined system we computed the INR using a Hocking-Mann simulation instead of the formula from [9]. Thus, for each individual case, new initial parameters were added, consisting of the initial levels of the species involved in the HM model. The levels of coagulation proteins were generated as independent Gaussian variables with a coefficient of variation as specified in table 1 (the median value was taken when multiple literature determinations were available). Two series of Monte Carlo simulations were run: (A) in which an average value of the baseline coagulation factor levels was taken to be the same for all cases in the random set of parameters, but other individual parameters (VKORC1, CYP2C9, antropometric differences) were kept variable; (B) in which the variabilities present in table 1 were also included in the baseline parameter generation.

## Results and Discussion

### Baseline factor levels vs INR

As previously detailed in the methods section, we first simulated the coagulation of normal blood samples using a stochastic version of the Hockin-Mann extrinsic coagulation pathway model.

**Figure 1:**
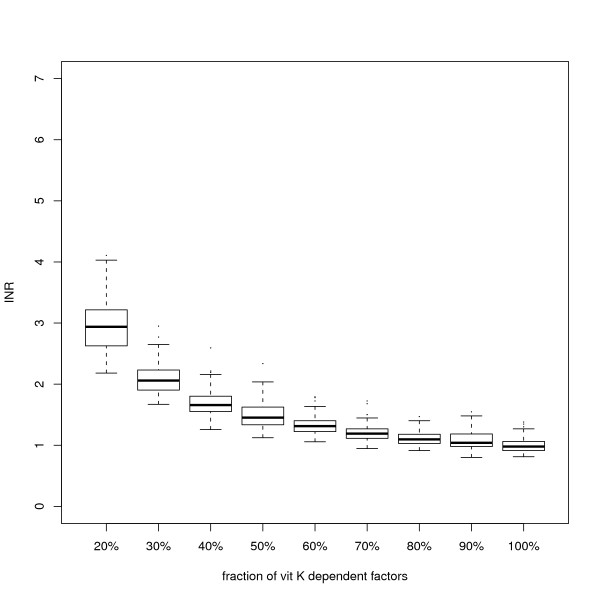
**Variability of the simulated INR by treatment intensity**. Each boxplot represents the distribution of INR values (reaction times relative to normal average) in a set of simulations. The biological levels of all coagulation proteins have been specified with truncated Gaussian distributions, taking the Hockin-Mann default values as means and applying the coefficient of variation from the literature reports in humans (table 1) to compute the standard deviation. The vitamin-K dependent factors were then reduced to the level specified on the *x *axis, corresponding to progressive treatment intensities. Horizontal lines in boxes represent the first, second and third quartiles. Dots represent outliers. The figure shows that the variability of the INR values increases when the levels of the vitamin-K dependent factors are decreasing.

Figure 1 shows the distribution of the simulated INR values at various levels of the vitamin K dependent factors (II, VII, IX and X), following different treatment intensities. The baseline level (100%) simulated sample resulted in a standard deviation of the INR of 0.12.

For the highest treatment intensity, corresponding to an average INR of about 3, despite reducing the levels--and implicitly the dispersion--of the vitamin K dependent factors to 20% the standard deviation of the INR increased to 0.48.

This result suggests that measuring the baseline levels of coagulation factors before treatment initiation might help with predicting the patient's sensitivity. As such measurements would be quite expensive, it is important to see which factor levels have the strongest association with the INR.

**Figure 2:**
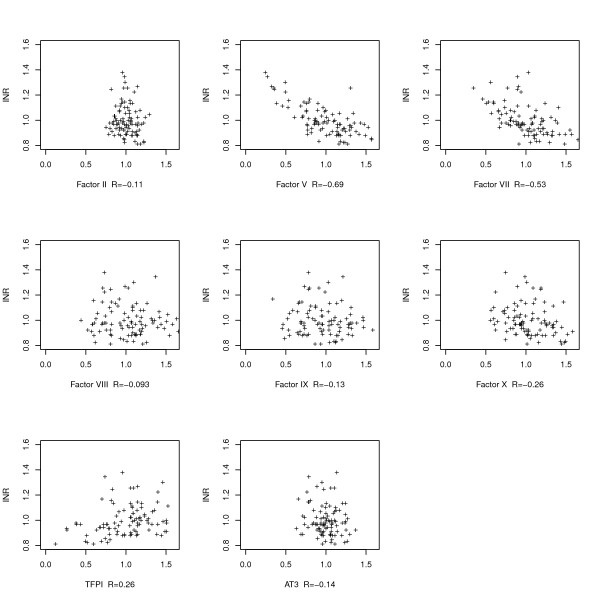
**Individual factor levels vs the INR in scenarios representing untreated subjects**. Vitamin-K dependent factors are at the baseline (pretreatment) level. R is the Pearson correlation coefficient for each plot. Each cross represents one point in the parameter space. The plot shows that factors V and VII have the strongest correlations with the baseline INR.

**Figure 3:**
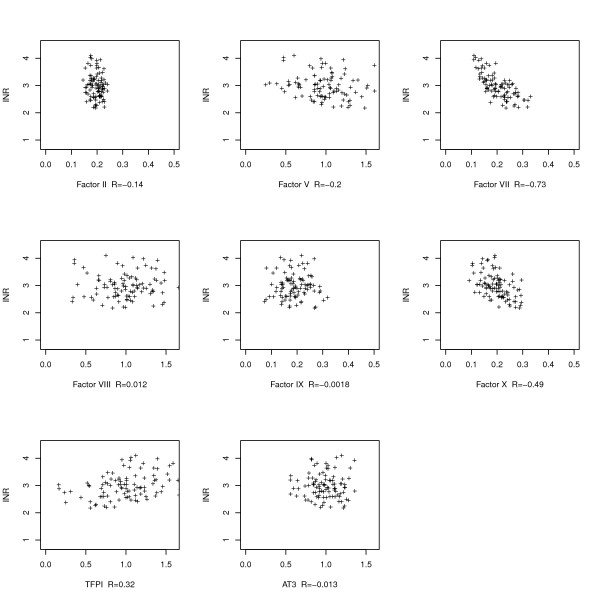
**Individual factor levels vs the INR in scenarios representing treated subjects**. Vitamin-K dependent factors are at 20% of the baseline level. Same conventions as in figure 2. Factors VII, X, and TFPI appear to have the strongest correlation with the INR.

Figures 2 and 3 show the relationship between the simulated level of each coagulation protein and the level of the INR in the healthy and treated scenarios, respectively.

Proconvertin and proaccelerin (factor VII and V) were important determinants of the INR value in untreated models. In the treated models factor V levels were not correlated with the INR value anymore, but factors VII, X and the TFPI were.

### Baseline factor levels and the warfarin dose

We combined our previously developed model of the population response to warfarin [11] with the Hockin-Mann model for computing the instantaneous INR values.

We ran two series of Monte Carlo simulations: one (series A) in which the normal variability of both the pharmacokinetic/pharmacodynamic (PK/PD) and the factor variability were represented and a second (series B) in which the PK/PD variability was represented but all baseline levels of the coagulation proteins were the same in all cases.

For each case in each Monte Carlo series we searched for the optimal daily dose of Warfarin for a target INR.

For a therapeutic INR target of 2.5, the dose in the population was 4.03 ± 1.67 mg/day in series B and 3.88 ± 2.00 mg/day in series A. Variances were 2.79 and 4.00; thus elimination of baseline variability of coagulation proteins led to a reduction of the simulated dose variance by 30%.

Table 2 shows the correlation coefficients between the baseline levels of the coagulation proteins proteins and the dose. Correlations with the dose were also stronger for factors VII, X and the TFPI.

**Table 2:** Correlation coefficients (R) of optimal warfarin dose vs. baseline coagulation protein levels

protein	R
Factor II	0.09
Factor V	0.07
Factor VII	0.32
Factor X	0.24
Factor VIII	0.09
TFPI	-0.30
AT-III	-0.01

The strongest correlations were found for factors VII, X and the tissue factor pathway inhibitor (TFPI).

### Discussion

Biological activity levels of plasma proteins involved in coagulation have been documented to exhibit a wide variability in normal subjects (see references from table 1). The fact this variability does not translate into an easily measurable variability of coagulation test results, such as the INR, that are based on measuring coagulation times in standardised conditions, is known in biology as "canalization" [5].

As a physiologic parameter, the optimal blood coagulation time is determined by the balance of the need to keep the blood flowing through all the blood vessels as long as the organism is intact and the need for it to clot following any wounding. Over the time of the evolution, the elimination of organisms that do not meet these two simultaneous and opposite constraints may have amounted to a "stabilizing selection" process that resulted in the canalization effect.

Despite its low effect on the INR, the normal variability of the baseline levels of some of these proteins (II, VII, TFPI, VIII, von Willebrand factor) has been associated with the risk of cardiovascular events such as myocardial infarction and stroke [12,13]. This illustrates how a "canalized" effect, such as the normally low variability of the coagulation time, can hide variabilities in other dimensions that are relevant for pathological variability.

Our results suggest a similar effect may occur with the response to anticoagulant treatment: while pretreatment coagulation times exhibit a low variability, the underlying variability of the coagulation protein levels may be related to clinically relevant differences in treatment response.

In this simulation study we explored the relationship between the variability of the pretreatment plasmatic factor levels and the variability of the coagulation time. We showed that the canalization effect should also be reduced when oral anticoagulation of the same intensity is applied to all the simulated patients, unmasking the effect of interindividual pretreatment factor variability on the INR only for patients under treatment. We also show that, when warfarin doses are adjusted such that the same INR target is achieved, the interindividual variability of pretreatment coagulation protein levels in the blood should result in an interindividual variability of the necessary dose.

These factors are influenced by a variety of known genetic polymorphisms [14-16] but the influence of each polymorphism is relatively small and also there are other, important, phenotypical and transient influences [17,18]. Thus, determination of their baseline biological levels rather than their genotype should be most effective as INR response predictors. Assays used for accurately measuring coagulation protein levels in the blood are currently quite expensive; however, if the possible association described here is confirmed experimentally more practical pretreatment investigations, such as coagulation tests complementary to INR determination, might be developed.

## Limitations

A limitation of our study is that we only considered a kinetic model of the extrinsic pathway of the coagulation cascade. Although this model was extensively validated with in vitro experimental data over the years, the particular implications we anticipated in this study need direct experimental verification as well.

The predictive value of the baseline coagulation protein levels may be lower than we expect as in our model we considered their synthesis and elimination processes stable in time for a given individual. The intraindividual variability [17,18] as well as inaccuracies of factor level measurement methods [19] may result in an actually lower predictive value.

## Conclusion

Our simulations suggest that in theory, to the extent of the validity of the Hockin-Mann model and our warfarin population effect model, the pretreatment levels of the coagulation proteins, in particular of factors X, VII and the TFPI may contribute to the variability of the response to warfarin. If experimental verification confirms our numeric predictions, measurement of the baseline levels of these proteins may further improve warfarin dose prediction.

We suspect that this type of effect --an unmasking of interindividual variability when some canalization effect is overcome--might also affect other types of treatments and pathological conditions and explain other causes of unexpected variability of the individual response.

## List of abbreviations

TFPI: tissue factor pathway inhibitor; AT-III: antithrombin III; II-XII: coagulation factors II to XII; HM: Hockin-Mann model (as described in reference 4); PK/PD: pharmacokinetic/pharmacodynamic; VKORC1: Vitamin K epoxyde reductase complex subunit 1; CYP2C9: Cytochrome P450 2C9; GGCX: Vitamin K dependent γ-glutamyl carboxylase; OAT: oral anticoagulant treatment; sd: standard deviation; c.v.: coefficient of variation; cvd: cardiovascular disease.

## Competing interests

The authors declare that they have no competing interests.

## Authors' contributions

ADC and JR designed the simulation experiments and wrote the paper. ADC performed the simulations and processed the results. All authors read and approved the final manuscript.
